# The use of laparoscopic surgery in the treatment of rectal cancer

**Published:** 2010-11-25

**Authors:** N Rîşnoveanu, AC Dima, M Ciurea

**Affiliations:** Emergency University Hospital, BucharestRomania

**Keywords:** laparoscopic surgery, rectal cancer, mesorectal excision

## Abstract

The use of laparoscopic techniques in the treatment of rectal cancer was until recently regarded with skepticism, because it did not seem to fulfill the oncologic principles of open surgery. The first report of a rectal cancer case treated by using laparoscopy has been made approximately two decades ago. From that moment on, the laparoscopic technique, progressed thanks to the development of the optical devices that allowed the improvement of the laparoscopic image, as well as the progressive increase of experience of the surgical teams specialized in colorectal laparoscopic surgery. These advantages (the faster recovery of the bowel function, less postoperative pain, lower blood loss, decreased hospitalization period) make the laparoscopic surgery a viable option for the treatment of rectal cancer. Recently published studies prove the similar results between open surgery and laparoscopic surgery for rectal cancer, in terms of mortality and postoperative morbidity, local recurrence, long–term survival or postoperative complications (anastomotic fistulas, bladder dysfunction, and sexual dysfunction), some studies even revealing the superiority of the laparoscopic surgery in preserving the function of the pelvic nerves.

## Introduction

The laparoscopic surgery for colon cancer has developed relatively late, as the earliest cases of laparoscopic colon cancer resection have been reported in 1991.[1,2] However, in the last two decades, the laparoscopic surgery of colon cancer has greatly progressed. Comparative and randomized studies made on significant groups of patients have shown similar results for laparoscopic and open colon cancer surgery, regarding the safety of the resection margins, tumor recurrence and long–term survival.[3,4,5,6]

However, despite the success obtained by using laparoscopic techniques in the treatment of colon cancer, the laparoscopic surgery for the treatment of rectal cancer implies many more difficulties (for exposing the pelvis, rectal dissection, sphincter preservation).[7,8, 9]

Generally, the conversion rate on open surgery is greater for rectal cancer, in comparison with colon cancer; therefore, in some studies, rectal cancer has even been considered a risk factor for the conversion to open surgery.[10,11,12] In addition to this, the conversion from laparoscopic to open surgery in the case of rectal cancer has been associated in some studies with the growth of the morbidity rate (probably because of the alteration of the immunological status).[13,14] New information from the literature does not support this fact.[15]

All the advantages brought by laparoscopic surgery: earlier recovery of bowel function, less postoperative pain, reduced blood loss, decreased hospital stay, faster social reintegration, support the use of laparoscopic intervention in the treatment of rectal cancer.[16,17,18,19]

## Aspects regarding the laparoscopic surgery of rectal cancer

The treatment of rectal cancer has considerably progressed in the last years, both because of the practice of complete mesorectal excision and the wider scale usage of neoadjuvant therapy for the local advanced disease.

In fact, the biggest concern regarding the laparoscopic resection of rectal cancer should be to perform a correct complete mesorectal excision, respecting the principles of sharp surgical dissection that would prevent a possible incomplete dissection (the risk of blunt dissection).

The neoadjuvant therapy, radiotherapy (cobalt therapy) with/without systemic chemotherapy for locally advanced disease (T3, T4) and N positive stages, used for decreasing the cancer stage [20], has already become a standard in the treatment protocols of rectal cancer.

Data presented in the first published studies regarding laparoscopic surgery of rectal cancer referred only to the laparoscopic abdominoperineal resection of tumors from the lower third of the rectum, because this was a technically easier surgery to perform. [21,22,23] Thus, it avoided the rectal resection under the tumor with the possibility of tumor–positive resection margins and the performing of colorectal (or coloanal) mechanical anastomosis with the implicit risk of anastomotic fistula. The abdominoperineal resection procedure was easier to complete due to the perineal time of the intervention that was done by using the classical method. Afterwards, the improved optics of the laparoscopic devices along with the special training of the surgeons in practicing rectal laparoscopic surgery, led to performing lower anterior rectal resection with complete mesorectal excision and colorectal intracorporeal anastomosis with very good results.[6,8,24,25,26]

The laparoscopic intervention must follow the same oncological principles as the open surgery. The standardization of the surgical technique is made according to the tumor's location. For tumors located in the upper third of the rectum, the procedure is done by resecting the tumoral rectum along with the excision of the mesorectum to approximately 5 cm distal from the macroscopic inferior margin of the tumor, which is considered safe from an oncological point of view.  For tumors located in the middle or the lower third of the rectum, a common procedure is the ablation of the tumoral rectum, along with the total excision of the mesorectum, with or without coloanal anastomosis. In this case, a limit of 2 cm of the distal resection from the tumor is considered safe.[15] For very low–situated tumors, when the anal sphincter must be preserved, and, in order to respect the oncological principles of resection at the same time, the best procedure is to perform the intersphincterial resection followed by coloanal transphincterial anastomosis.[27] In the cases of low and very low–situated anastomosis, when the risk of detachment from the anastomosis cannot be overlooked, a protective ileostoma should be made, that would be subsequently eliminated.[28]

Retrospective studies, that have demonstrated the reduction of the local recurrence and improvement of the survivability for the patients with rectal cancer who have had their mesorectum completely excised, have imposed this stage as mandatory in the surgical treatment of rectal cancer. Thus, the most important stage in the laparoscopic surgery of rectal cancer is the correct removal of the mesorectum, by respecting the principles of dissection described in the classical surgery, and, at the same time, trying to preserve the pelvic nerves by avoiding postoperative urinary and sexual complications.[29,30] The positive intraperitoneal pressure generated by the pneumoperitoneum, induced in order to perform the surgical intervention, creates a good dissection plane of the mesorectum, as it opens up the alveolar plane of natural cleavage, that separates the parietal and visceral fascia.[31]

Only a few studies were carried to compare the results regarding the oncological safety of the resection margins (circumferential and distal), as it is a well–known fact that positivity resection margins are an independent oncological prognosis factor for rectal cancer. The CLASSIC Trial has highlighted a greater percent in the positivity of resection margins after laparoscopic surgery, in comparison with the lower anterior resection made through open surgery (12% vs. 6%).[13] Other studies, conducted in centers specialized in laparoscopic surgery of rectal cancer, have not shown differences regarding the positivity of resection margins obtained after laparoscopic surgery versus open surgery.[19,32,33]

This year, Laurent C et al have published the results of the largest study created in order to compare the results from a distance between laparoscopic and open surgery of the rectal cancer. The study was done on 471 patients, operated between 1997 and 2006, of which 80% presented lower and median rectal tumors, most of them being locally advanced. The study results did not show any difference between laparoscopic and open surgery of the rectal cancer regarding the average appearance time of the local recurrence (16.9 months vs 15.9 months; p=0.827), local recurrence after 5 years (3.9% vs 5.5%; p=0.371), metastasis presence after 5 years (20.6% vs 24.9%; p=0.415).[15] 

All the studies that have directly compared laparoscopic and open surgery of the rectal cancer have not showed differences between the two, regarding morbidity and mortality (morbidity between 6.1 and 40%; mortality between 0 and 3%).[31]

In spite of the minimally invasive approach, a reduction of the pulmonary and cardiovascular complications has not been demonstrated for patients who had a laparoscopic procedure.[6,34]

The total resection of the mesorectum by using laparoscopy should ensure a better preservation of sexual and urinary bladder functions, due to a better view of the pelvic nerves.[25,35] The urinary bladder dysfunction is reported to be at around 0–12%, and the sexual dysfunction at around 10–35% for the patients who have undergone laparoscopic rectal resection. There are no significant statistical differences in comparison with open surgery.[36,37,38]

The average time reported for the laparoscopic resection of rectal cancer has been generally longer than for the open surgery–between 165 and 260 minutes.[8,9,20,26,32–34,39–43] However, shorter times have been reported for laparoscopic surgery in comparison with the open surgery (228 min vs 284 min; p=0.04).[44,45] These data show that the experience of the surgical team is vital in determining the total time.

   The advantages of laparoscopic surgery in comparison with the open surgery for rectal cancer are: lower blood loss (between 78 ml and 320 ml) [8,24,26,32,33,39,42], faster bowel recovery (3–5 days postoperatively)[20,24,32,40–43], faster introduction of a normal diet (3–6 days postoperatively) )[20,24,32,40–43],shorter hospital stay after the laparoscopic intervention (8–11 days). [8,9,20,26,32–34,39–43] 

The impossibility of a tumor resection by using laparoscopy implies the conversion to open surgery. In case of a hybrid intervention for a rectal cancer, when some of the operating times use laparoscopy (dissection of the inferior mesenteric blood vessels, mobilization of the splenic flexure and the left–side colon), and others, partially use open surgery (the total excision of the mesorectum, tumor extraction), the conversion to open surgery is difficult to define. In this case, conversion to open surgery is defined in different ways by a series of authors: any abdominal incision greater than 7 cm (Braga et al)[33], vertical abdominal incision which is larger than the tumor that has to be excised (CLASICC)[34], any unplanned abdominal incision necessary for finalizing the intervention, in order to control the haemostasis or the rectal mobilization (Kim et al)[19], the need for a median line conversion (Laurent et al)[46], the interruption of the laparoscopic procedure (Staudacher et al).[24]

The causes that determine the conversion from the laparoscopic approach in rectal cancer to the open surgery are a locally advanced tumor, obesity, adhesion, impossibility of rectal resection, difficulties in creating an anastomosis, organ lesion, impossibility of tumor localization, hemorrhage.[47,48,49]

The largest study, conducted on 1073 patients, that followed the impact of the conversion to open surgery in rectal cancer, concluded that turning to open surgery resulted in substantially higher morbidity, in comparison with the complete laparoscopic procedure (54% for patients converted to open surgery compared with 24% for patients with complete laparoscopic procedure).[14]

The most frequent complications that appeared during surgery, to patients who require conversion to open surgery were: impossibility of rectal resection because it was fixed and it had not been observed on the preoperative images (11.5%), impossibility of completing the anastomosis (8.9%), organ damage (inferior mesenteric artery, rectum, small intestine, ureter, hypogastric nerve, posterior vaginal wall) (7.7%), and hemorrhage (3.8%). For patients who underwent a complete laparoscopic rectal resection, the most frequent complications were: organ damage (1.1%), hemorrhage (1.0%), difficulties in creating anastomosis (0.7%) and rectal resection (0.4%).[14]

In this study conducted in Japan, the conversion rate was lower, of only 7.3%, in comparison with the results reported in previous studies in western countries, where the conversion rate was of about 15%.[50–52] The difference noticed in the two regions may be explained by: the lower number of obese patients in the Asian population, selection of patients in early stages of the disease, lack of routine–applied neoadjuvant therapy. 

In another study, however, there was no difference between the postoperative morbidity in patients who had a conversion to open surgery, compared with patients with a finalized laparoscopic intervention (16.7% vs 23.8%; p=0.349) or the long-term impact: local recidive after 5 years (3.5% vs 3.8%; p=0.739) distance recurrence in 5 years (19.4% vs 19.9%; p=0.466), survival in 5 years (91% vs 83%; p=0.350).[15]

It is considered to be very important to create an ‘early’ conversion on patients who are considered difficult to be treated by laparoscopy before critical complications could appear.[10,53]

## Conclusions

The more numerous studies have been published in the specialty literature in the last years regarding the oncological safety of applying laparoscopic surgery in the treatment of rectal cancer, the more they did not show differences regarding the rate of local recidive and the distance survival between laparoscopic and open surgery. The anatomic–pathological studies made on resection pieces after the laparoscopic surgeries on rectal cancer showed similar results regarding the safety of circumferential and distal resection margins. There are still a lot of question marks about this new chapter in surgery, though the results of randomized controlled multicentered trials (COLORII, ACOSOG–Z6051) now in progress, and which will probably offer some of the answers. In the future, laparoscopic surgery for rectal cancer should become a safe means of treatment for selected patients with rectal cancer.
